# The changing characteristics of a cohort of children and adolescents living with HIV at antiretroviral therapy initiation in Asia

**DOI:** 10.1371/journal.pone.0291523

**Published:** 2023-09-14

**Authors:** Johanna Beulah Sornillo, Rossana Ditangco, Aarti Kinikar, Dewi Kumara Wati, Quy Tuan Du, Dinh Qui Nguyen, Vohith Khol, Lam Van Nguyen, Thanyawee Puthanakit, Pradthana Ounchanum, Nia Kurniati, Kulkanya Chokephaibulkit, Thahira A. Jamal Mohamed, Tavitiya Sudjaritruk, Siew Moy Fong, Nagalingeswaran Kumarasamy, Pope Kosalaraksa, Revathy A. Nallusamy, Nik Khairulddin Nik Yusoff, Annette H. Sohn, Azar Kariminia, on behalf of the TREAT Asia Pediatric HIV Observational Database of IeDEA Asia-Pacific

**Affiliations:** 1 Department of Epidemiology and Biostatistics, Research Institute for Tropical Medicine, Manila, Philippines; 2 Medical Department, Research Institute for Tropical Medicine, Manila, Philippines; 3 BJ Medical College and Sassoon General Hospital, Pune, India; 4 Department of Pediatrics, Sanglah Hospital, Udayana University, Bali, Indonesia; 5 Infectious Diseases Department, Children’s Hospital 1, Ho Chi Minh City, Vietnam; 6 Infectious Diseases Department, Children’s Hospital 2, Ho Chi Minh City, Vietnam; 7 National Center for HIV/AIDS, Dermatology and STD, Phnom Penh, Cambodia; 8 Infectious Diseases Department, National Hospital of Pediatrics, Hanoi, Vietnam; 9 Department of Pediatrics, Faculty of Medicine and Research Unit in Pediatric and Infectious Diseases, Chulalongkorn University, Bangkok, Thailand; 10 Department of Pediatrics, Chiangrai Prachanukroh Hospital, Chiang Rai, Thailand; 11 Faculty of Medicine Universitas Indonesia, Cipto Mangunkusumo, Jakarta, Indonesia; 12 Department of Pediatrics, Faculty of Medicine, Siriraj Hospital, Mahidol University, Bangkok, Thailand; 13 Pediatric Institute, Women and Children Hospital Kuala Lumpur, Kuala Lumpur, Malaysia; 14 Department of Pediatrics, Faculty of Medicine, Research Institute for Health Sciences, Chiang Mai University, Chiang Mai, Thailand; 15 Department of Pediatrics, Hospital Likas, Kota Kinabalu, Malaysia; 16 VHS-Infectious Diseases Medical Centre, Chennai, India; 17 Department of Pediatrics, Faculty of Medicine, Khon Kaen University, Khon Kaen, Thailand; 18 Department of Pediatrics, Penang Hospital, Penang, Malaysia; 19 Department of Pediatrics, Hospital Raja Perempuan Zainab II, Kota Bharu, Malaysia; 20 TREAT Asia, amfAR—The Foundation for AIDS Research, Bangkok, Thailand; 21 The Kirby Institute, UNSW Sydney, Sydney, New South Wales, Australia; University of Cape Town School of Public Health and Family Medicine, SOUTH AFRICA

## Abstract

Despite improvements in HIV testing and earlier antiretroviral therapy (ART) initiation in children living with HIV through the years, a considerable proportion start treatment with advanced disease. We studied characteristics of children and adolescents living with HIV and their level of immunodeficiency at ART initiation using data from a multi-country Asian cohort. We included children and adolescents who were ART-naïve and <18 years of age at ART initiation from 2011 to 2020 at 17 HIV clinics in six countries. Incidence rates of opportunistic infections (OIs) in the first two years of triple-drug ART (≥3 antiretrovirals) was also reported. Competing risk regression analysis was performed to identify factors associated with first occurrence of OI. In 2,027 children and adolescents (54% males), median age at ART initiation increased from 4.5 years in 2011–2013 to 6.7 in 2017–2020, median CD4 count doubled from 237 cells/μl to 466 cells/μl, and proportion of children who initiated ART as severely immunodeficient decreased from 70% to 45%. During follow-up, 275 (14%) children who received triple-drug ART as first treatment and had at least one clinic visit, developed at least one OI in the first two years of treatment (9.40 per 100 person-years). The incidence rate of any first OI declined from 12.52 to 7.58 per 100 person-years during 2011–2013 and 2017–2020. Lower hazard of OIs were found in those with age at first ART 2–14 years, current CD4 ≥200 cells/μl, and receiving ART between 2017 and 2020. The analysis demonstrated increasing number of children and adolescents starting ART with high CD4 count at ART start. The rate of first OI markedly decreased in children who started ART in more recent years. There remains a clear need for improvement in HIV control strategies in children, by promoting earlier diagnosis and timely treatment.

## Introduction

In 2022, an estimated 930,000 children aged less than 10 years and 1.65 million adolescents aged 10–19 years were living with HIV worldwide [1]. In that year, there were an estimated 270,000 children and adolescents with newly acquired HIV. Although countries have been placing more people on HIV treatment over the past decade, globally, only 57% of children aged 0–14 years and 65% of adolescents aged 10–19 years were receiving treatment in 2022 compared with 77% of adults [1–3]. Starting on antiretroviral therapy (ART) early is key to optimizing the health of people living with HIV (PLHIV) to achieve viral suppression and improved health outcomes, as opposed to the consequences of late presentation to care and delayed treatment [4, 5].

Since 2004, the World Health Organization (WHO) has regularly issued evidence-based guidelines for HIV treatment informed by expert opinion and community values and preferences. In 2010, WHO recommended ART initiation for all HIV-positive infants <24 months of age, regardless of CD4 level [6]. This was extended to all children <5 years of age in 2013 [7]. In 2016, WHO recommended starting all PLHIV on ART, regardless of their clinical or immune status [5]. Current international and local HIV management guidelines encourage immediate initiation of ART after HIV diagnosis, except for patients with specific opportunistic infections (OIs) for which immediate ART is contraindicated [8].

While there have been clear improvements in decreasing age, increasing CD4 count, and reduced disease severity at ART initiation in children and adolescents living with HIV [9–13], a substantial proportion still present with advanced symptomatic disease. In a global analysis conducted in 32 countries between 2004 to 2013, more than 40% of children in low- and middle-income countries and less than 20% in high income countries had severe immunodeficiency at ART initiation by 2013 [14]. In the Asia Pacific, most countries have expanded ART programs in line with WHO treatment recommendations. UNAIDS estimated that treatment coverage of children and younger adolescents (0–14 years) living with HIV in the region was 76% in 2021, higher than the 66% among adults [15]. However, progress has been uneven across countries. For example, pediatric HIV treatment coverage reported in 2021 was higher than 95% in Malaysia, but only 56% in Cambodia and 25% in Indonesia. Other countries in the region also reported relatively higher coverage at 75% in Thailand, 82% in Vietnam, and 95% in India [16]. Moreover, there have been concerning declines in maternal ART coverage in the region following the COVID-19 pandemic, falling from 58% in 2019 to 49% in 2021, which poorly compares to the 81% global average [1, 2]. With only half of pregnant women with HIV receiving ART for their health and to prevent vertical transmission, efforts to achieve elimination of pediatric HIV will stagnate or fail.

We used data from a regional longitudinal pediatric HIV cohort to describe characteristics of children and adolescents and their degree of immunodeficiency at ART initiation from 2011 to 2020, and assess whether improvements in ART coverage over time have resulted in decline in the incidence of OIs. This information is needed to further improve care and program strategies and, in turn, pediatric HIV treatment outcomes in the region.

## Materials and methods

### Study population

The TREAT Asia pediatric HIV Observational Database (TApHOD) is an observational clinical cohort of the International epidemiology Databases to Evaluate AIDS (IeDEA), and its methodology has been previously reported [17]. Briefly, TApHOD enrolls children and adolescents seen at 17 clinics in Cambodia (n = 1), India (n = 2), Indonesia (n = 2), Malaysia (n = 4), Thailand (n = 5) and Vietnam (n = 3). These sites are pediatric referral clinics within larger healthcare facilities (n = 13) or freestanding pediatric hospitals (n = 4). Demographic and clinical data are collected from medical records at each site, de-identified, and sent to the Kirby Institute, University of New South Wales, for aggregation, quality assessment and processing. Only site principal investigators (site physicians) could identify their patients during or after data collection. The database includes information on over 7400 children and adolescents who received care at the participating sites from 2001 to 2020. In this analysis, we included all children and adolescents (except for exposure to perinatal prophylaxis), who were ART-naïve and <18 years of age at ART initiation between 1 January 2011 and 31 December 2020. We used all available follow-up data up to December 2020.

Approval for the study was granted by the human research ethics committees at all participating sites, the data management and analysis center at the Kirby Institute (UNSW Sydney), and the coordinating center at TREAT Asia/amfAR (The Foundation for AIDS Research). Consent by parents or legal guardians and assent of the children and adolescents under care were not routinely obtained, unless required by the local ethics committee (i.e., in some sites in India, Malaysia, and Thailand).

### Outcomes and key variable definitions

The primary outcomes analyzed were trends in patient characteristics at ART initiation (age, CD4 count, WHO clinical stage, and weight-for-age z score [WAZ]). The secondary outcome was incidence of first OI event within the first two years of ART. OI events were defined on the basis of diagnoses recorded in the medical records at each site. Adolescence were defined as 10 to 19 years of age, and children defined as <10 years. Children and adolescents who were enrolled younger than 15 years and have no other documented mode of infection reported were defined as perinatally acquired HIV. For laboratory and clinical measurements at treatment initiation, we used the closest values reported during a testing window of six months before to one week after ART start for CD4 and HIV viral load, and a window of six months before to four weeks after for WAZ, with priority given to the pre-ART measurement. For WHO clinical stage, we used the highest stage reported from six months before through the date of ART initiation. We defined severe HIV-associated immunodeficiency according to WHO criteria as the CD4 levels less than 25% (age <1 year), less than 20% (age 1 to <3 years), less than 15% (age 3 to 5 years), and less than 15% or less than 200 cells per mm^3^ (age ≥5 years) [18]. We converted weight measurements to WAZ using 2006 WHO standards [19] for children ≤10 years or Centers for Disease Control and Prevention standards [20] for children >10 years. We divided the year of ART start into three time periods (2011 to 2013, 2014 to 2016, and 2017 to 2020) to correspond to changes in WHO treatment guidelines.

### Statistical analysis

We used descriptive statistics (number and percentage, median and interquartile range [IQR]) to summarize participants’ characteristics at ART initiation and by year of ART initiation (i.e., 2011–2013, 2014–2016, 2017–2020). In a subset of patients who had received at least three antiretrovirals as their initial treatment regimen and who had at least one follow-up visit after starting ART, we calculated the incidence rates of OI per 100 person-years (with 95% confidence intervals [CIs]) for the first occurrence of any OI, and for the first occurrence of each individual OI in the first two years of ART. The incidence rate was obtained by dividing the number of first OIs by person-years of observation and reported separately among patients who started ART in each time period. Follow-up was from the start of ART and ceased at the earliest of the following: the date of the OI being analyzed (i.e., first occurrence of any OI or the first occurrence of a specific OI), date of death, date of most recent visit, day prior to the patient’s 20^th^ birthday, and at two years. To assess factors associated with the first occurrence of any OI, we used Fine and Gray’s subdistribution hazard model [21] to account for the competing effect of death. This model evaluates the effect of an exposure on the cause-specific hazard of the outcome of interest, while accounting for the presence of competing events. The following variables were included in the univariate analysis: sex, age, WAZ, severe immunodeficiency, and time period at first triple-drug ART, facility level, and country income group. We included CD4 count as a time-updated variable and if a CD4 count was missing, the previous CD4 count was carried forward. We did not use multiple imputation to replace missing data because there were relatively small numbers of covariates available. We also considered clinic as a random effect in the model. The initial multivariate model included all covariates with a P value of <0.20 on univariate analysis. We then used a backward stepwise approach and retained covariates with a P value of <0.05 in the final model. Crude and adjusted subdistribution hazard ratios (asHR) with 95% CI were reported as the measures of association. Missing data were included in the regression analyses as a separate category within each variable. The proportional hazards assumption was assessed using the estat phtest command in Stata. Data were analyzed using Stata version 12 (Stata Corporation, College Station, Texas, USA) and SAS Software (Version 9.4 for Windows).

## Results

**Fig 1:**
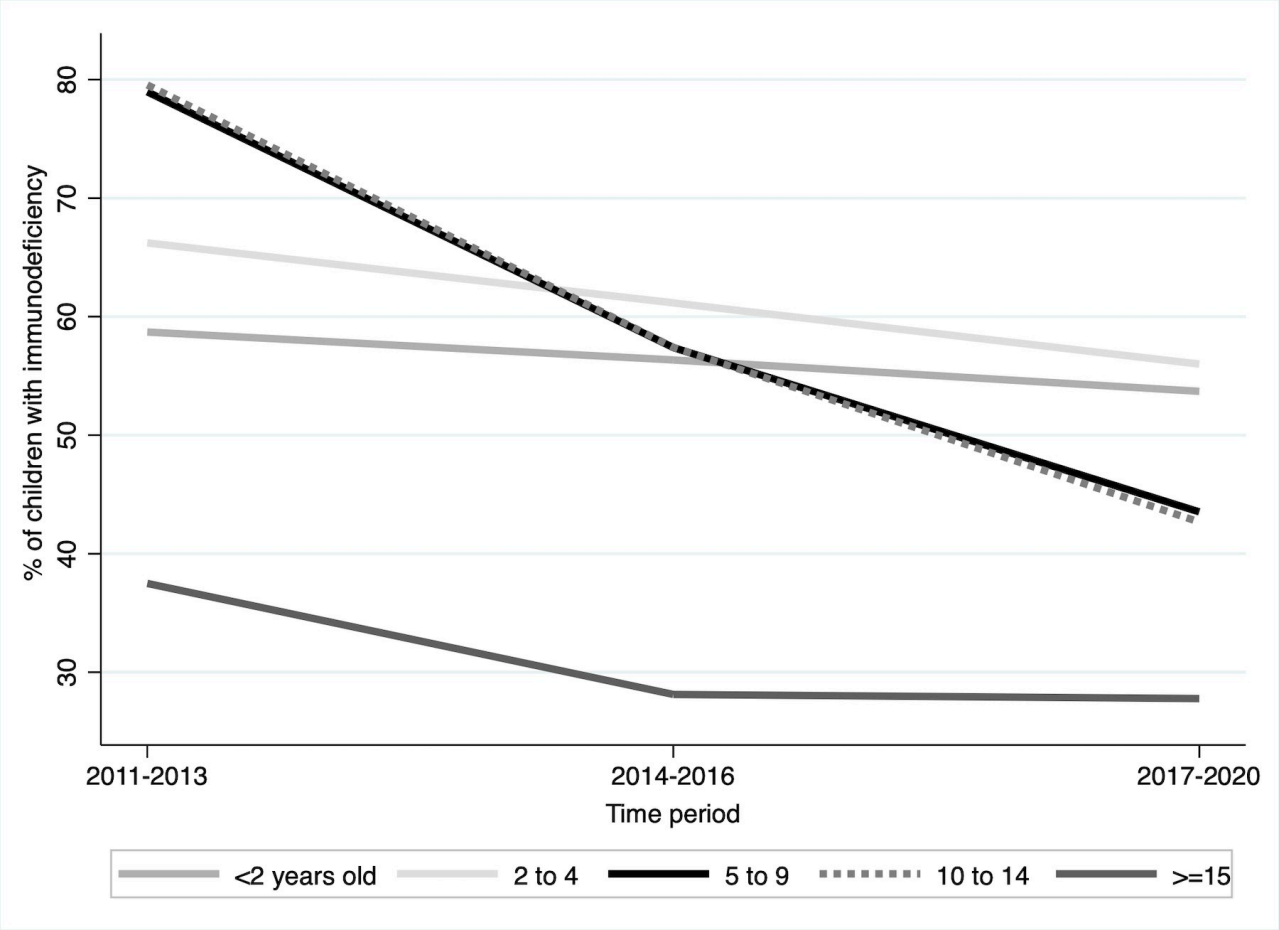
Proportion of patients with severe immunodeficiency at ART start, by age group and calendar time period at ART start (n = 2027).

A total of 2027 (54% males) children and adolescents were eligible for inclusion in the analysis. Patient characteristics at first ART are summarized by period of first ART in Table 1. Median age at ART initiation increased from 4.5 years (IQR 1.7–7.9) in 2011–2013 to 6.7 (IQR 2.1–11.3) in 2017–2020, mainly driven by the increasing proportion of adolescents which was doubled over the same period. Overall, 56% of younger adolescents (10–14 years) and 60% of older adolescents (15–19 years) were male, with male accounting for 76% of older adolescents in 2017–2020. The percentage of children and adolescents with an available CD4 count at ART initiation declined over time from 87% in 2011–2013 to 64% in recent years. From 2011 to 2020, there was a doubling in median CD4 count at ART initiation overall from 237 cells/μl in 2011–2013 to 466 cells/μl in 2017–2020, and proportion of patients initiating ART with CD4 ≥ 500 cells/μl rose from 34% to 46%. Overall, there was a decrease in the proportion of patients who initiated treatment with severe immunodeficiency from 70% for those who initiated in 2011–2013 to 45% for those who initiated in 2017–2020. Among different age groups, the highest decrease in the proportion with severe immunodeficiency was observed in the age groups 5–9 years (79% to 43%) and 10–14 years (79% to 42%) and the lowest decrease was observed in the age group <2 years (59% to 52%) (Fig 1). Overall, half of the children and adolescents had advanced HIV disease, and the proportion of those who were severely underweight at ART start has remained largely unchanged (31%) over this period.

**Table 1:** Characteristics at ART start by period of ART initiation.

	Total	2011–13	2014–16	2017–20
**Patients (n)**	2027	747	739	541
**World Bank Country income group**				
Upper-middle income	302 (15)	114 (15)	103 (14)	85 (16)
Lower-middle income	1725 (85)	633 (85)	636 (86)	456 (84)
**Sex**				
Male	1100 (54)	401 (54)	407 (55)	292 (54)
Female	927 (46)	346 (46)	332 (45)	249 (46)
**Age, years**				
Median (IQR)	5.2 (1.8–9.9)	4.5 (1.7–7.9)	5.9 (1.9–10.1)	6.7 (2.1–11.3)
<2	531 (26)	211 (28)	191 (26)	129 (24)
2 to 4	447 (22)	201(27)	147 (20)	99 (18)
5 to 9	569 (28)	219 (29)	212 (29)	138 (26)
10 to 14	372 (18)	98 (13)	145 (20)	129 (24)
≥15	108 (5.3)	18 (2.4)	44 (6.0)	46 (8.5)
**CD4 count, cells/μl**				
Available data	1597 (79)	652 (87)	598 (81)	347 (64)
Median (IQR)	338 (71–793)	237 (39–752)	355 (117–838)	466 (141–809)
<200	616 (39)	313 (48)	199 (33)	104 (30)
200–349	194 (12)	63 (9.7)	98 (16)	33 (9.5)
350–499	162 (10)	56 (8.6)	57 (9.5)	49 (14)
≥500	625 (39)	220 (34)	244 (41)	161 (46)
**Median CD4 count by age (IQR)**				
<2 years	1108 (533–1848)	1099 (495–1697)	1140 (604–1839)	1108 (458–2232)
Available data	379 (71)	170 (81)	144 (75)	65 (50)
2 to 4	454 (114–843)	379 (54–687)	608 (193–906)	527 (186–1086)
Available data	373 (83)	179 (89)	124 (84)	70 (71)
5 to 9	142 (26–411)	61 (22–243)	226 (49–452)	398 (35–398)
Available data	463 (81)	195 (89)	180 (85)	88 (64)
10 to 14	180 (40–373)	103 (16–211)	204 (49–353)	355 (97–578)
Available data	290 (78)	91 (93)	114 (79)	85 (66)
≥15	351 (197–501)	239 (163–527)	293 (134–406)	391 (229–519)
Available data	92 (85)	17 (94)	36 (82)	39 (85)
**Severe HIV-associated immunodeficiency**				
Available data	1493 (74)	611 (82)	561 (76)	321 (59)
No	611 (41)	186 (30)	247 (44)	178 (55)
Yes	882 (59)	425 (70)	314 (56)	143 (45)
**HIV viral load, copies/ml**				
Available data	332 (16)	131 (18)	91 (12)	110 (20)
Median log10 (IQR)	5.4 (4.8–6.0)	5.5 (5.1–6.1)	5.4 (4.7–6.1)	5.1 (4.6–5.9)
400–999	5 (1.5)	1 (0.8)	0	4 (3.6)
1,000–9,999	26 (7.8)	6 (4.6)	9 (9.9)	11 (10)
≥10,000	301 (91)	124 (95)	82 (90)	95 (86)
**Weight-for-age z-score**				
Available data	1783 (88)	684 (92)	623 (84)	476 (88)
Median	-2.2 (-3.4 to -1.1)	-2.3 (-3.4 to -1.2)	-2.3 (-3.4 to -1.0)	-2.1 (-3.2 to -1.1)
<-3	585 (33)	232 (34)	207 (33)	146 (31)
-3≤ to <-2	396 (22)	160 (23)	139 (22)	97 (20)
-2≤ to <-1	398 (22)	143 (21)	126 (20)	129 (27)
≥-1	404 (23)	149 (22)	151 (24)	104 (22)
**WHO clinical stage**				
Available data	1230 (61)	588 (79)	387 (52)	255 (47)
Stage I/II	544 (44)	251 (43)	168 (43)	125 (49)
Stage III	498 (40)	255 (43)	153 (40)	90 (35)
Stage IV	188 (15)	82 (14)	66 (17)	40 (16)
**Initial ART regimen**				
NNRTI-based	1587 (78)	648 (87)	587 (79)	352 (65)
PI-based	362 (18)	70 (9.4)	133 (18)	159 (29)
INSTI-based	33 (1.6)	2 (0.3)	5 (0.7)	26 (4.8)
NRTI	37 (1.8)	26 (3.5)	11 (1.5)	0
Mono/dual	8 (0.4)	1 (0.1)	3 (0.4)	4 (0.7)

Data are n (%), median (IQR, interquartile range). For weight-for-age z-score, the 2006 WHO standards was used for children ≤10 years and the Centers for Disease Control and Prevention standards for children >10 years. ART, antiretroviral therapy; NNRTI, non-nucleoside reverse transcriptase inhibitor; PI, protease inhibitor; INSTI, integrase strand transfer inhibitors; NRTI, nucleoside reverse transcriptase inhibitor; mono/dual, single or two drugs.

**Fig 2:**
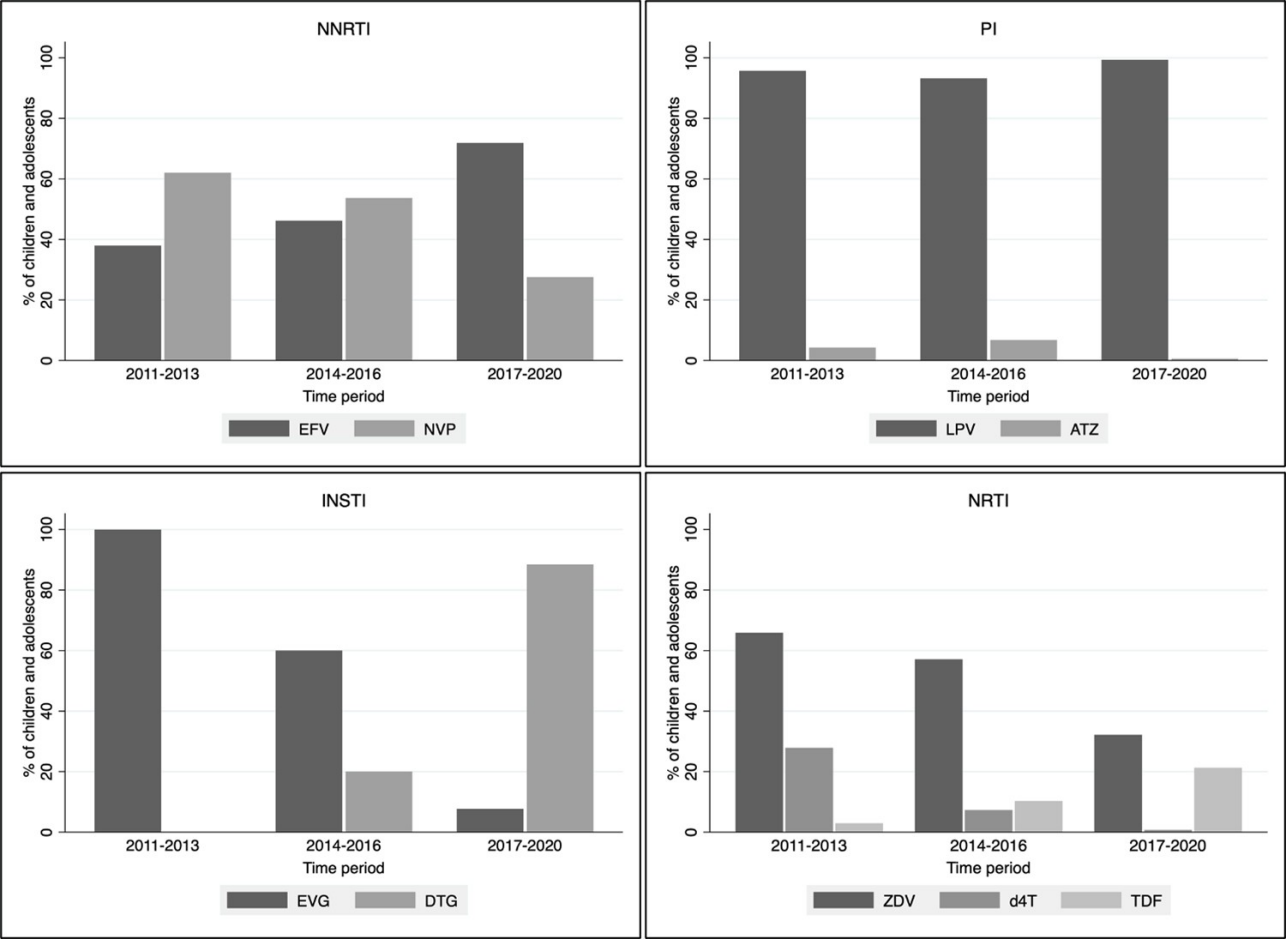
Proportion of children and adolescents on ART who used specific drug or drug combination by calendar period of initiation and drug class (n = 2019). NNRTI = non-nucleoside reverse transcriptase inhibitor; PI = protease inhibitor; INSTI = integrase strand transfer inhibitor; NRTI = nucleoside reverse transcriptase inhibitor; EFV = efavirenz; NVP = nevirapine; LPV = lopinavir/ritonavir; ATZ = atazanavir/ritonavir; EVG = elvitegravir; DTG = dolutegravir; ZDV = zidovudine; d4T = stavudine; TDF = tenofovir.

Between 2011 and 2020, most children and adolescents started a non-nucleoside reverse transcriptase inhibitor (NNRTI)-based regimen, but the use of NNRTIs decreased as protease inhibitor (PI)-based regimens increased after 2015 (Table 1). Efavirenz replaced nevirapine as the most commonly used NNRTI (from 38% in 2011–2013 to 72% in 2017–2020). The NRTIs zidovudine and stavudine were replaced by abacavir- and tenofovir-containing first-line regimen (Fig 2). There was negligible (<1%) use of a first-line integrase strand transfer inhibitor (INSTI)-based regimen prior to 2017, but 4.8% of patients received a INSTI-based initial regimen from 2017 to 2020. For the majority (92%) of patients, first INSTI-based contained Elvitegravir.

### Incidence of OIs

Of 2027 children and adolescents, 1963 who had received triple-drug ART as their initial treatment and who had at least one clinic visit after starting ART were included in the OI analysis. Of the 64 excluded, two were on mono or dual therapy only, six were exposed to mono or dual therapy before starting triple-drug ART, and 56 did not have a clinic visit after starting ART (of which eight died, 18 transferred, 14 were lost to follow-up or LTFU, and 13 were not recorded as died, transferred, or LTFU). During 2011–2020, the total period of observation contributing to the first two years of ART was 3298 person-years with a median follow-up period of 2.00 years (IQR 1.68–2.00). Of 1963 children and adolescents, 275 experienced at least one OI resulting in a crude incidence rate of any first OI of 9.40 (95% CI 8.35–10.58) per 100 person-years (Table 2). When observation was restricted to the first year of ART only, the crude incidence rate of first OI was 13.95 (95% CI 12.25–15.90) per 100 person-years. The incidence rate of any first OI per 100 person-years was 12.52 in 2011–2013, 7.48 in 2014–2016, and 7.58 in 2017–2020. Recurrent upper respiratory tract infection and pulmonary tuberculosis (TB) remained the leading OIs, irrespective of the calendar period of starting ART, with an overall incidence of 4.26 (95% CI 3.59–5.05) and 1.39 (95% CI 1.04–1.86) per 100 person-years, respectively. These two infections and other common OIs, including severe recurrent bacterial pneumonia and lymphoid interstitial pneumonitis appeared much less common in children and adolescents who first received ART after 2014.

**Table 2:** Incidence rate per 100 person-years of the first occurrence of OIs and death in the first two years of ART, overall and by calendar time period of ART start.

OIs	All	2011–2013	2014–2016	2017–2020
**Number of patients**	1963	740	707	516
**All OIs**	402	223	113	66
Person-years	3298	1310	1252	736
Incidence (95% CI)	12.19 (11.05–13.44)	17.02 (14.93–19.41)	9.02 (7.50–10.85)	8.97 (7.05–11.42)
**Any first OI**	275	138	85	52
Person-years	2925	1101	1137	686
Incidence (95% CI)	9.40 (8.35–10.58)	12.52 (10.60–14.80)	7.48 (6.05–9.25)	7.58 (5.77–9.94)
**Deaths**	69	42	22	5
Person-years	3826	1415	1385	1027
Incidence (95% CI)	1.80 (1.42–2.28)	2.97 (2.19–4.02)	1.59 (1.05–2.41)	0.49 (0.20–1.17)
**First specific OI, WHO clinical stage 2**				
Recurrent upper respiratory tract infection	133	79	39	15
Person-years	3124	1197	1205	722
Incidence (95% CI)	4.26 (3.59–5.05)	6.60 (5.29–8.23)	3.24 (2.36–4.43)	2.08 (1.25–3.45)
Extensive wart virus infection,	11	8	2	1
Person-years	3282	1299	1249	735
Incidence (95% CI)	0.34 (0.19–0.61)	0.62 (0.31–1.23)	0.16 (0.04–0.64)	0.14 (0.02–0.97)
**First specific OI, WHO clinical stage 3**				
Pulmonary tuberculosis	45	22	16	7
Person-years	3236	1273	1235	729
Incidence (95% CI)	1.39 (1.04–1.86)	1.73 (1.14–2.63)	1.30 (0.79–2.12)	0.96 (0.46–2.01)
Severe recurrent bacterial pneumonia	28	19	6	3
Person-years	3256	1279	1243	735
Incidence (95% CI)	0.86 (0.59–1.25)	1.49 (0.95–2.33)	0.48 (0.22–1.07)	0.41 (0.13–1.27)
Lymph node tuberculosis	20	13	4	3
Person-years	3269	1290	1246	733
Incidence (95% CI)	0.61 (0.39–0.95)	1.01 (0.58–1.73)	0.32 (0.12–0.86)	0.41 (0.13–1.27)
Lymphoid interstitial pneumonitis	24	12	10	2
Person-years	3265	1294	1239	732
Incidence (95% CI)	0.73 (0.49–1.10)	0.93 (0.53–1.63)	0.81 (0.43–1.50)	0.27 (0.07–1.09)
Unexplained persistent diarrhoea	11	4	2	5
Person-years	3285	1304	1249	732
Incidence (95% CI)	0.33 (0.19–0.60)	0.31 (0.12–0.82)	0.16 (0.04–0.64)	0.68 (0.28–1.64)
Persistent oral candidiasis	10	3	5	2
Person-years	3279	1304	1243	732
Incidence (95% CI)	0.30 (0.16–0.57)	0.23 (0.07–0.71)	0. 40 (0.17–0.97)	0.27 (0.07–1.09)
**First specific OI, WHO clinical stage 4**				
Pneumocystis pneumonia	15	3	6	6
Person-years	3278	1304	1242	731
Incidence (95% CI)	0.46 (0.28–0.76)	0.23 (0.07–0.71)	0.48 (0.22–1.08)	0.82 (0.37–1.83)
Extrapulmonary or disseminated tuberculosis	14	5	3	6
Person-years	3282	1304	1247	731
Incidence (95% CI)	0.43 (0.25–0.72)	0.46 (0.21–1.02)	0.24 (0.08–0.75)	0.68 (0.28–1.64)
Cytomegalovirus retinitis or cytomegalovirus infection	6	1	2	3
Person-years	3289	1308	1248	733
Incidence (95% CI)	0.18 (0.08–0.41)	0.08 (0.01–0.54)	0.16 (0.04–0.64)	0.41 (0.13–1.27)
Oesophageal candidiasis	2	2	0	0
Person-years	3294	1306	1252	736
Incidence (95% CI)	0.06 (0.02–0.24)	0.15 (0.04–0.61)	0	0
Disseminated mycobacteriosis	3	0	1	2
Person-years	3293	1310	1250	733
Incidence (95% CI)	0.09 (0.03–0.28)	0	0.08 (0.01–0.57)	0.27 (0.07–1.09)
Disseminated mycosis	2	0	0	2
Person-years	3295	1310	1252	733
Incidence (95% CI)	0.07 (0.02–0.24)	0	0	0.27 (0.07–1.09)

95% CI, 95% confidence interval.

Risk factors for developing any first OIs in the first two years of ART start are shown in Table 3. In the final fitted multivariate model, lower hazard rates of OI were associated with age at first ART 2–14 years compared to <2 years (lowest asHR for 10–14 years 0.48 95% CI 0.28–0.82), current CD4 ≥200 cells/μl compared to <200 cells/μl (lowest asHR for 350–499 cells/μl 0.19 95% CI 0.10–0.37 and ≥500 cells/μl 0.19 95% CI 0.12–0.31), and receiving ART between 2017 and 2020 compared with between 2011 and 2013 (asHR 0.46 95% CI 0.22–0.97). Receiving care in regional, provincial, or university hospitals was found to be associated with an increased risk of developing first OI (vs. health centers; asHR 3.53 95% CI 1.37–9.07).

**Table 3:** Characteristics related to development of first OI within the first two years of ART start (where mortality is a competing event).

Characteristics	Total (n = 1963)	Follow-up time (person-years)	First OI (n = 275)	Univariate analysis		Multivariate analysis^c^	
				*SHR (95% CI)*	*p-value*	*aSHR (95% CI)*	*p-value*
**Sex**					0.415		
Male	1059	1606	144	1			
Female	904	1319	131	1.08 (0.90–1.31)			
**Age at first ART (years)**					0.007		0.001
<2	515	741	96	1		1	
2–4	437	676	60	0.71 (0.49–1.02)		0.59 (0.38–0.90)	
5–9	559	847	75	0.70 (0.52–0.94)		0.50 (0.34–0.72)	
10–14	353	502	40	0.62 (0.31–1.24)		0.48 (0.28–0.82)	
≥15	99	159	4	0.20 (0.04–1.05)		0.27 (0.05–1.35)	
**Current CD4 count (cells/ul)** ^**a**^					<0.001		<0.001
<200		258	89	1		1	
200–349		219	16	0.28 (0.19–0.42)		0.25 (0.18–0.36)	
350–499		304	14	0.21 (0.11–0.38)		0.19 (0.10–0.37)	
≥500		2009	118	0.29 (0.18–0.48)		0.19 (0.12–0.31)	
Missing data		135	38	0.99 (0.52–1.88)		0.93 (0.53–1.63)	
**Weight-for-age z-score at first ART**					0.014		*0*.*422*
<-3	558	737	95	1			
-3 ≤ to <-2	391	598	57	0.81 (0.64–1.02)		*1*.*00 (0*.*80–1*.*23)*	
≥-2	786	1249	99	0.68 (0.48–0.96)		*0*.*85 (0*.*64–1*.*13)*	
Missing data	228	340	24	0.59 (0.24–1.47)		*0*.*82 (0*.*34–1*.*96)*	
**Severe HIV-associated immunodeficiency**					<0.001		*0*.*463*
No	591	975	48	1			
Yes	866	1275	149	2.23 (1.67–2.98)		*1*.*17 (0*.*80*–*1*.*71)*	
Missing data	506	674	78	2.13 (1.46–3.11)		*1*.*02 (0*.*61*–*1*.*71)*	
**Year of ART start** ^**b**^					0.299		0.124
2011–2013	740	1102	138	1		1	
2014–2016	707	1137	85	0.64 (0.35–1.15)		0.66 (0.40–1.06)	
2017–2020	516	686	52	0.58 (0.25–1.33)		0.46 (0.22–0.97)	
**Facility level**					0.026		0.009
Healthcare center	781	1129	48	1		1	
Regional, provincial, or university hospital	1182	1796	227	3.09 (1.14–8.39)		3.53 (1.37–9.07)	
**Country income group**					0.606		
Lower-middle income	1671	2456	244	1			
Upper-middle income	292	469	31	0.70 (0.18–2.74)			

OI, opportunistic infection. Death (n = 45) was a competing event for first OI in this analysis. Total numbers include missing values. Missing values were included as a separate category in all analyses. Global p values have excluded missing categories. 95% CI, 95% confidence interval; asHR, adjusted subdistribution hazard ratio.

^a^ CD4 count was considered time-dependent variable. Total number was not given as children and adolescents moved between categories.

^b^ Year of ART start was our variable of interest and was included in the final model, even if non-significant in the univariate analysis.

^c^ Adjusted for current CD4 count, age at ART start, year of ART start, and facility level (competing risks regression analysis with death as competing event); Weight-for-age z-score at first ART and severe HIV-associated immunodeficiency were not remained in the multivariate model. Their aSHR (shown in italics) were obtained by adding and removing them individually to the final model.

## Discussion

In this study involving children and adolescents who started ART in the Asia region, we describe demographic and clinical characteristics at ART start across time periods. Between 2011 and 2020, the absolute numbers of children starting treatment decreased in the cohort, with improvements in the median CD4 counts at ART start and decline in the overall proportions with severe immunodeficiency. By 2017 to 2020, an increasing proportion of patients with CD4 cell counts >500 cells/μl were receiving early treatment. Furthermore, children and adolescents initiating ART after 2013 had almost half the risk of experiencing an OI in the first two years of treatment compared to those initiating ART in 2011–2013, and mortality decreased substantially.

The decline in the number and proportion of children <5 years at ART start may reflect local progress in implementing effective vertical prevention strategies in some of the countries represented in the cohort. In Asia, Thailand and Malaysia met WHO criteria for elimination of mother-to-child transmission (MTCT) of HIV in 2016 and 2018, respectively, with transmission rates of <2% [22, 23]. Reduction of infant transmission to less than 5% has also been reported in recent years in Cambodia [24] and Vietnam [25]. Furthermore, our study clinics are mainly located within tertiary facilities and the decentralization of HIV care to local primary care clinic may have also contributed to the overall reduction in the absolute number of children initiating ART [26]. In our cohort, there were more males in the older adolescent group, which was consistent with the recent report of UNAIDS in East Asia and Pacific where male adolescents were two times more likely than females to contract HIV. This pattern is different with sub-Saharan Africa, where adolescent females were three times more likely to contract HIV than males [27]. In general, evidence suggests that male adolescents are more likely to engage in risky behavior than their female counterparts, and in many countries in the Asia Pacific region, gender norms encourage early onset of sexual behaviour and high number of sexual partners. Together with inconsistent condom use, this could explain the high risk of HIV in male adolescents in the region [28]. Along with the two-fold increase in the proportion of younger adolescents and the three-fold increase in the proportion of older adolescents over the study period, their median CD4 count at ART start also increased from 2011–2013 and reached up to over 350 cells/μl in 2017–2020. This finding is consistent with that of Apondi et al who has shown similar increase in the proportion of older adolescents at ART start in their study in East Africa [29]. Implementation of the Treat All strategy, rise in HIV testing and linkage to care, education on the AIDS epidemic, and counselling are described by the authors as likely contributing factors to this increase [30, 31].

High proportions of children and adolescents living with HIV were severely immunosuppressed in our study (70% in 2011–2013 and 45% in 2017–2020) and we observed a modest decrease in the proportion of children <5 years of age who were immunodeficient (62% to 55%). Given the greater risk of rapid disease progression and worse HIV outcomes in children <5 years of age compared to older children [32], this finding highlights the need to ensure earlier diagnosis and ART initiation for infants with perinatally acquired HIV. In a previous global analysis, 42% to 64% of children <16 years living in low- and middle-income countries who received treatment in 2013 were severely immunodeficient [14]. In a more recent study, 55% of children <5 years in 2014–2017 started treatment with severe immunodeficiency [12]. Notably, an IeDEA study conducted in earlier years (2004–2013) found only a small decline in the proportion of children <1 year with severe immunodeficiency over time [14]. Likewise, the small decrease observed in the proportion of severely immunosuppressed in children <2 years from 2011–2013 to 2017–2020 could be partly explained by late diagnosis and delayed and poor engagement in care among those diagnosed. Poor access to and low coverage of MTCT in lower-middle income countries may explain the small reduction we observed in very young children. Indonesia is an example, where despite national efforts, only 28% of pregnant women were tested for HIV in 2020 [33].

In this study, 14% of children experienced at least one OI during the first two years of ART. Recurrent upper respiratory tract infection and pulmonary TB were the most common OIs over the study period. We observed a small decline in the incidence of TB over time. This is consistent with data from studies in sub-Saharan Africa demonstrating the ongoing risk of HIV-associated TB even among populations with high ART coverage [34, 35]. HIV infection increases the progression of TB infection to active disease and worsens disease severity [36]. This makes it essential that HIV programs strengthen their existing strategies of early detection of TB and monitoring the development of both diseases. In the multivariate analyses, higher hazard rate of OI is observed if ART is started at <2 years compared to initiating at 2 years or older. This can possibly attributed to the higher incidence of some of the OIs in the younger children, including recurrent or chronic upper respiratory tract infection (5.81 per 100 person-years in <2 years vs. 3.43 per 100 person-years in 2–14 years).

We used data from clinical settings collected through the course of routine care, there is the potential for incomplete and/or inconsistent data, which limits the reliability of our findings when applied to broader populations of children and adolescents with HIV. Notably, the shift to treat-all policies that are not reliant on CD4 counts has meant that there are fewer children starting ART with baseline CD4 tests. This is compounded by the variability in available methods for diagnosing OIs. Moreover, TApHOD sites are mainly university-based clinics and/or referral centers located in capital cities. While they represent the major pediatric HIV care and treatment centers in their geographic areas, the sites may not be fully representative of the greater population of children and adolescents living with HIV across the region. In addition, residual confounding in the multivariable analyses due to unmeasured covariates is of concern in observational studies and may have affected our findings.

## Conclusions

Our analyses suggest that across the three time periods examined, there have been marked increases in the median CD4 count at ART initiation in our regional cohort, and substantial improvement in health outcomes, as measured by persistent reductions in the incidence rates of mortality and first OIs. An increasing proportion of children and adolescents did not have a baseline CD4 count, and a substantial proportion were still initiating treatment with severe immunodeficiency. Despite some of the recent successes in pediatric HIV control in the region, there is a clear need for further program enhancement, with earlier diagnosis and immediate treatment for children.

## Supporting information

S1 Checklist STROBE statement—checklist of items that should be included in reports of observational studies. (DOCX)

S1 Fig Flow diagram of the selection of the study sample. (TIFF)

S1 Table Number and proportion of all OIs experienced in the first two years of ART (n = 1963). (DOCX)
