# Supplementary material for: The changing characteristics of a cohort of children and adolescents living with HIV at antiretroviral therapy initiation in Asia
Source: PLoS One. 2023 Sep 14;18(9):e0291523. doi: 10.1371/journal.pone.0291523 (PMC10501581; doi:10.1371/journal.pone.0291523)
Supplement: S1 Checklist — (DOCX) [file pone.0291523.s001.docx]

STROBE Statement—checklist of items that should be included in reports of observational studies

|  | Item No. | Recommendation | Page  No. | Relevant text from manuscript |
| --- | --- | --- | --- | --- |
| **Title and abstract** | 1 | (*a*) Indicate the study’s design with a commonly used term in the title or the abstract | 1 | Lines 2-3  “The changing characteristics of a cohort of children and adolescents living with HIV at antiretroviral therapy initiation in Asia” |
|  |  | (*b*) Provide in the abstract an informative and balanced summary of what was done and what was found | 3 | Lines 54-74  *An abstract is provided with the following: background, materials and methods, results, and discussion and conclusion.* |
| Introduction | | | |  |
| Background/rationale | 2 | Explain the scientific background and rationale for the investigation being reported | 4-5 | Lines 80-115  In the Asia Pacific region, the treatment coverage of children and younger adolescents (0-14 years) living with HIV in the Asia Pacific region was 76% in 2021 according to the UNAIDS, which is higher than the 66% among adults. To our knowledge, there has been no published studies to describe the characteristics of these children at ART initiation over time. |
| Objectives | 3 | State specific objectives, including any prespecified hypotheses | 5 | Lines 117-121  We described characteristics of children and adolescents and their level of immunodeficiency at ART initiation from 2011 to 2020, and assessed whether improvements in ART coverage over time have resulted in the decrease in incidence of OIs. |
| Methods | | | |  |
| Study design | 4 | Present key elements of study design early in the paper | 6 | Lines 129-131  This is an observational clinical cohort study using data from the TREAT Asia pediatric HIV Observational Database (TApHOD). |
| Setting | 5 | Describe the setting, locations, and relevant dates, including periods of recruitment, exposure, follow-up, and data collection | 6 | Lines 131-142  Our study population includes children and adolescents under care between January 2001 to December 2020 in 17 TApHOD clinics in Cambodia (n=1), India (n=2), Indonesia (n=2), Malaysia (n=4), Thailand (n=5) and Vietnam (n=3). These sites are pediatric referral clinics within larger healthcare facilities (n=13) or freestanding pediatric hospitals (n=4). Follow-up of children started from ART initiation after enrolment until the last visit. |
| Participants | 6 | (*a*) *Cohort study*—Give the eligibility criteria, and the sources and methods of selection of participants. Describe methods of follow-up  *Case-control study*—Give the eligibility criteria, and the sources and methods of case ascertainment and control selection. Give the rationale for the choice of cases and controls  *Cross-sectional study*—Give the eligibility criteria, and the sources and methods of selection of participants | 6 | Lines 129-142  This study is an observational cohort. TApHOD enrolls all children and adolescents with confirmed HIV diagnosis. In this study, we included children who received ART since 1^st^ of January 2011 after enrollment into clinic. |
|  |  | (*b*) *Cohort study*—For matched studies, give matching criteria and number of exposed and unexposed  *Case-control study*—For matched studies, give matching criteria and the number of controls per case | - | *Not applicable* |
| Variables | 7 | Clearly define all outcomes, exposures, predictors, potential confounders, and effect modifiers. Give diagnostic criteria, if applicable | 6-7 | Lines 152-169  The outcomes studied were characteristics at ART initiation and incidence of first OI within 2 years of triple-drug ART.  The variables included in the analysis were the following: age, sex, clinical and laboratory characteristics (e.g., CD4 count, viral load), year of ART start, and facility characteristics. |
| Data sources/ measurement | 8* | For each variable of interest, give sources of data and details of methods of assessment (measurement). Describe comparability of assessment methods if there is more than one group | 6 | Lines 134-138  Data has been provided by data manager from the medical records. De-identified data were sent to the data management center for data quality checks and aggregation. |
| Bias | 9 | Describe any efforts to address potential sources of bias | 8 | Lines 192-195  Because this was an observational cohort study, selection bias could be an issue. However, this has been partly addressed by controlling for baseline characteristics (confounders) in the regression analysis. |
| Study size | 10 | Explain how the study size was arrived at | - | Sample size was not calculated for this study**.** We have included all children who attended the participating clinics.  In our sample of 1963 children, we are able to detect a hazard ratio of 0.07 with a power higher than 90%. |

Continued on next page

| Quantitative variables | 11 | Explain how quantitative variables were handled in the analyses. If applicable, describe which groupings were chosen and why | 7; 8 | Lines 158-169; 186-190  We have treated the following quantitative variables as fixed, namely, sex, age at ART initiation, WAZ, severe immunodeficiency, time period at ART initiation, facility level, setting, and country income group. CD4 count was included as a time-updated variable.  We calculated WAZ using WHO standards for children < 10 years and CDC for > 10 years. We have categorized CD4 and viral load according to routine and clinical practice (for the former, <200 cells/mm^3^, 200-349, 350-499, >500 cells/mm^3^). WHO criteria was used as basis for categorizing HIV-associated immunodeficiency. We considered the timing of the release of WHO treatment guidelines for the time period. |
| --- | --- | --- | --- | --- |
| Statistical methods | 12 | (*a*) Describe all statistical methods, including those used to control for confounding | 7-8 | Lines 172-199  The statistical methods used are detailed under ‘Statistical analysis’, where the use of competing risk analyses based on Fine and Gray’s proportional sub-hazards model was also described. |
|  |  | (*b*) Describe any methods used to examine subgroups and interactions | - | *Not applicable* |
|  |  | (*c*) Explain how missing data were addressed | 8 | Lines 188-191; 195-196  We carried forward previous CD4 count if a value is missing. For time-updated CD4 count, WAZ at first ART, severe immunodeficiency, and WHO clinical stage, we included missing data in the regression analysis as a separate category. |
|  |  | (*d*) *Cohort study*—If applicable, explain how loss to follow-up was addressed  *Case-control study*—If applicable, explain how matching of cases and controls was addressed  *Cross-sectional study*—If applicable, describe analytical methods taking account of sampling strategy | - | *Not applicable* |
|  |  | (*e*) Describe any sensitivity analyses | - | *Not applicable* |
| Results | | | | |
| Participants | 13* | (a) Report numbers of individuals at each stage of study—eg numbers potentially eligible, examined for eligibility, confirmed eligible, included in the study, completing follow-up, and analysed | S1 Fig | Refer to the consort diagram to demonstrate the selection of study sample. |
|  |  | (b) Give reasons for non-participation at each stage | S1 Fig | Refer to the same consort diagram to determine reasons for exclusion of some selected participants. |
|  |  | (c) Consider use of a flow diagram | S1 Fig 1 | We have used a consort diagram to report on the process of selection. |
| Descriptive data | 14* | (a) Give characteristics of study participants (eg demographic, clinical, social) and information on exposures and potential confounders | 9-12 | Refer to Table 1 (entitled ‘Characteristics at ART start by period of ART initiation’) |
|  |  | (b) Indicate number of participants with missing data for each variable of interest | 9-12 | Refer to Table 1 (entitled ‘Characteristics at ART start by period of ART initiation’), specifically for variables CD4 count, severe immunodeficiency, HIV viral load, weight-for-age z score, WHO clinical stage |
|  |  | (c) *Cohort study*—Summarise follow-up time (eg, average and total amount) | 14-18 | To calculate for the incidence of first OI and death, the follow-up times were 2925 and 3826 person-years, respectively. The follow-up time per time period and OI are detailed in Table 2 (entitled, ‘Incidence rate per 100 per years of the first occurrence of OIs and death in the first two years of ART, overall and by calendar time period of ART start’). |
| Outcome data | 15* | *Cohort study*—Report numbers of outcome events or summary measures over time | 9-12  14-18 | Table 1 records the summary and trends of patient characteristics, overall and per time period of ART initiation (2011-2013, 2014-2016, and 2017-2020). Table 2 reports the incidence rates of occurrence of any first OI diagnosed and deaths within two years, with rates of each 14 OIs (2 for WHO clinical stage 2, 6 for stage 3, 6 for stage 4). These are also reported overall and by period of ART initiation. |
|  |  | *Case-control study—*Report numbers in each exposure category, or summary measures of exposure | - | *Not applicable* |
|  |  | *Cross-sectional study—*Report numbers of outcome events or summary measures | - | *Not applicable* |
| Main results | 16 | (*a*) Give unadjusted estimates and, if applicable, confounder-adjusted estimates and their precision (eg, 95% confidence interval). Make clear which confounders were adjusted for and why they were included | 19-21 | Table 3 records both the crude and adjusted hazard ratios of the characteristics related to the development of first OI within first years of ART start, namely, sex, age at first ART, time-updated CD4 count, WAZ at first ART, severe immunodeficiency, year of ART start, facility level, and country income group. |
|  |  | (*b*) Report category boundaries when continuous variables were categorized | 9-12  14-18  19-21 | Category boundaries are presented in the tables (Tables 1-3). |
|  |  | (*c*) If relevant, consider translating estimates of relative risk into absolute risk for a meaningful time period | - | *Not applicable* |

Continued on next page

| Other analyses | 17 | Report other analyses done—eg analyses of subgroups and interactions, and sensitivity analyses | - | *Not applicable* |
| --- | --- | --- | --- | --- |
| Discussion | | | | |
| Key results | 18 | Summarise key results with reference to study objectives | 22-24 | Lines 299-362  The Discussion explains the trends in the demographic and clinical characteristics at ART start across time periods and how these results compare to data from other studies. |
| Limitations | 19 | Discuss limitations of the study, taking into account sources of potential bias or imprecision. Discuss both direction and magnitude of any potential bias | 24-25 | Lines 364-374  In the last part of Discussion, limitations in the generalizability of findings are discussed, including the presence of residual confounding due to covariates not included in the analysis. |
| Interpretation | 20 | Give a cautious overall interpretation of results considering objectives, limitations, multiplicity of analyses, results from similar studies, and other relevant evidence | 25 | Lines 381-388  Our analysis has shown substantial improvement in status and health outcomes of children and adolescents at ART initiation. Still, this reflects necessity to implement HIV control strategies by promoting earlier diagnosis and timely treatment. |
| Generalisability | 21 | Discuss the generalisability (external validity) of the study results | 24 | Lines 369-372  Because of the type of facilities enrolled in TApHOD which are, in general, major hubs of pediatric HIV care and treatment, those included in the analysis may not adequately represent majority of the pediatric population who reside in these areas in the region. |
| Other information | |  | | |
| Funding | 22 | Give the source of funding and the role of the funders for the present study and, if applicable, for the original study on which the present article is based | - | *Not included in the manuscript as instructed by PLOS ONE. The information below will be entered in the financial disclosure section of the submission system.*  “The TREAT Asia pediatric HIV Observational Database is an initiative of TREAT Asia, a program of amfAR, The Foundation for AIDS Research, with support from the US National Institutes of Health’s National Institute of Allergy and Infectious Diseases, the  Eunice Kennedy Shriver National Institute of Child Health and Human Development, National Cancer Institute, National Institute of Mental Health, National Institute on Drug Abuse, the National Heart, Lung, and Blood Institute, the National Institute on Alcohol Abuse and Alcoholism, the National Institute of Diabetes and Digestive and Kidney Diseases, and the Fogarty International Center, as part of the International epidemiology Databases to Evaluate AIDS (IeDEA; U01AI069907). The Kirby Institute is funded by the Australian Government Department of Health and Ageing, and is affiliated with the Faculty of Medicine, UNSW Australia. The content of this publication is solely the responsibility of the authors and does not necessarily represent the official views of any of the governments or institutions mentioned above. The funders had no role in study design, data collection and analysis, decision to publish, or preparation of the manuscript.” |

*Give information separately for cases and controls in case-control studies and, if applicable, for exposed and unexposed groups in cohort and cross-sectional studies.

**Note:** An Explanation and Elaboration article discusses each checklist item and gives methodological background and published examples of transparent reporting. The STROBE checklist is best used in conjunction with this article (freely available on the Web sites of PLoS Medicine at http://www.plosmedicine.org/, Annals of Internal Medicine at http://www.annals.org/, and Epidemiology at http://www.epidem.com/). Information on the STROBE Initiative is available at www.strobe-statement.org.
