# Supplementary figures and images for: The changing characteristics of a cohort of children and adolescents living with HIV at antiretroviral therapy initiation in Asia
Source: PLoS One. 2023 Sep 14;18(9):e0291523. doi: 10.1371/journal.pone.0291523 (PMC10501581; doi:10.1371/journal.pone.0291523)

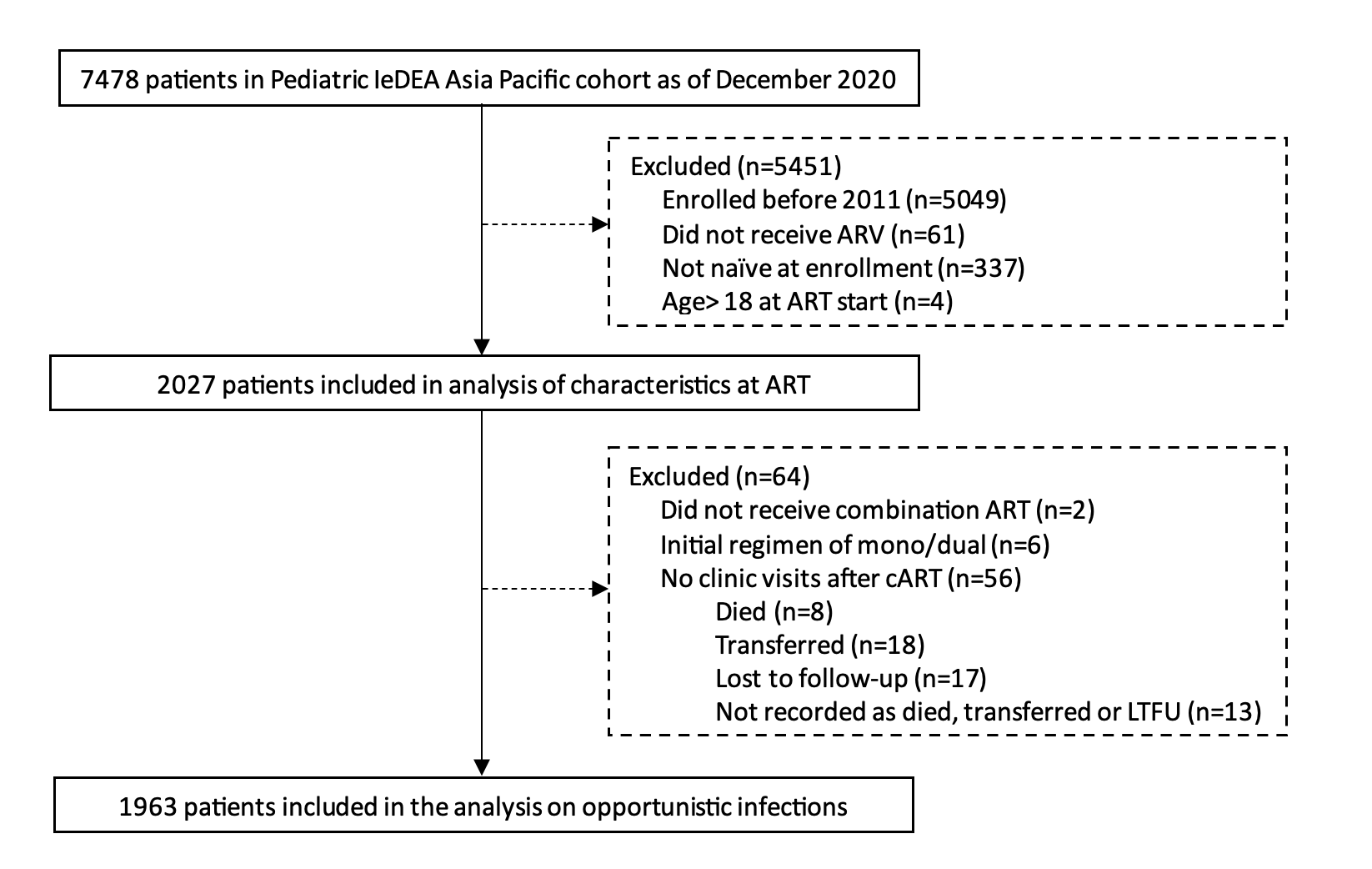

Supplement: S1 Fig — (TIFF) [file pone.0291523.s002.tiff]
