# Supplementary material for: The changing characteristics of a cohort of children and adolescents living with HIV at antiretroviral therapy initiation in Asia
Source: PLoS One. 2023 Sep 14;18(9):e0291523. doi: 10.1371/journal.pone.0291523 (PMC10501581; doi:10.1371/journal.pone.0291523)
Supplement: S1 Table — (DOCX) [file pone.0291523.s003.docx]

**S1 Table. Number and proportion of all OIs experienced in the first two years of ART (n=402)**

| **WHO events** | **n (%)** |
| --- | --- |
| Stage 2 |  |
| Extensive wart virus infection | 12 (3.0) |
| Extensive molluscum contagiosum | 2 (0.5) |
| Recurrent or chronic upper respiratory tract infections (otitis media, otorrhoea, sinusitis, tonsillitis) | 192 (47.8) |
| Fungal nail infections | 2 (0.5) |
| Stage 3 |  |
| Unexplained persistent diarrhoea (14 days or more) | 11 (2.7) |
| Persistent oral candidiasis (after first 6 weeks of life) | 10 (2.5) |
| Acute necrotizing ulcerative gingivitis/periodontitis | 0 |
| Lymph node TB | 22 (5.5) |
| Pulmonary TB | 47 (11.7) |
| Severe recurrent bacterial pneumonia | 30 (7.5) |
| Symptomatic lymphoid interstitial pneumonitis | 29 (7.2) |
| Stage 4 |  |
| Oesophageal candidiasis (or candidiasis of trachea, bronchi or lungs) | 2 (0.5) |
| Extrapulmonary cryptococcosis (including meningitis) | 1 (0.2) |
| Chronic cryptosporidiosis | 0 |
| Cytomegalovirus retinitis or cytomegalovirus infection affecting another organ, with onset at age older than one month | 6 (1.5) |
| Chronic herpes simplex infection (orolabial or cutaneous of more than one month’s duration or visceral at any site) | 0 |
| Chronic Isospora | 0 |
| Kaposi sarcoma | 0 |
| Extrapulmonary or disseminated tuberculosis | 14 (3.5) |
| Disseminated mycobacteriosis, other than tuberculosis | 3 (0.7) |
| Disseminated mycosis (coccidiomycosis, histoplasmosis or penicilliosis) | 2 (0.5) |
| Pneumocystis pneumonia | 15 (3.7) |
| Recurrent severe bacterial infection, such as empyema, pyomyositis, bone or joint infection or meningitis but excluding pneumonia | 1 (0.2) |
| Progressive multifocal leukoencephalopathy | 1 (0.2) |
| Central nervous system toxoplasmosis onset after age one month | 0 |
